# Supplementary material for: HLA-DRB1*14:54 Is Associated with Pulmonary Alveolar Proteinosis: A Retrospective Real-World Audit
Source: Biomedicines. 2023 Oct 27;11(11):2909. doi: 10.3390/biomedicines11112909 (PMC10669482; doi:10.3390/biomedicines11112909)
Supplement: Supplementary file 1 [file biomedicines-11-02909-s001.zip › biomedicines-2647246-supplementary.pdf]

biomedicines

MDPI

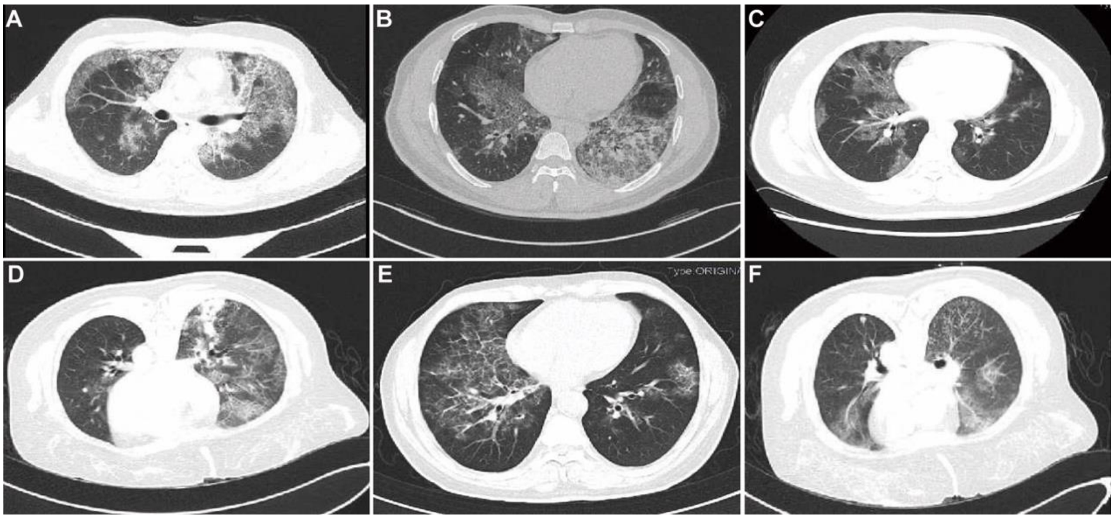

Supplementary Figure S1. Thoracic high-resolution computed tomography (HRCT) scans of patients with pulmonary alveolar proteinosis (PAP). HRCT typically reveals ground-glass opacification (GGO) in the lungs of PAP patients. (A-C) Bilateral GGO with sharp demarcation in patients with autoimmune PAP. (D-F) Diffuse GGO ± consolidation in patients with secondary PAP.

Supplementary Table 1. Primers for amplification and sequencing.

**Amplification primer**	**Sequence**
Forward primer	TGTAAAACGACGGCCAGTTTCTTGGAGTACTCTA
Reverse primer	CAGGAAACAGCTATGACCYGCTYACCTCGCCKCTGCAC
Sequencing primer	Sequence
Forward primer	TGTAAAACGACGGCCAGT
Reverse primer	CAGGAAACAGCTATGACC

Supplementary Table 2. HLA-DRB1 Genotypes frequencies.

**HLA-DRB1 genotypes**	**aPAP (N = 18), no. (%)**	**HLA-DRB1 genotypes**	**sPAP (N = 10), no. (%)**
08:03/14:54	4 (22.2)	15:01/16:02	2 (20.0)
08:03/14:05	1 (5.6)	08:03/10:01	1 (10.0)
13:12/14:54	1 (5.6)	08:03/11:01	1 (10.0)
09:01/10:01	1 (5.6)	08:03/13:01	1 (10.0)
04:05/08:03	1 (5.6)	09:01/12:01	1 (10.0)
09:01/14:54	1 (5.6)	09:01/15:01	1 (10.0)
03:01/08:03	1 (5.6)	03:01/09:01	1 (10.0)
01:01/04:03	1 (5.6)	04:05/15:01	1 (10.0)
08:03/13:12	1 (5.6)	12:02/15:01	1 (10.0)
15:01/16:02	1 (5.6)		
13:12/16:02	1 (5.6)		
11:06/12:02	1 (5.6)		
14:54/15:01	1 (5.6)		

**HLA-DRB1 genotypes**	**aPAP (N = 18), no. (%)**	**HLA-DRB1 genotypes**	**sPAP (N = 10), no. (%)**
07:01/08:03	1 (5.6)		
11:01/16:02	1 (5.6)		

aPAP = autoimmune pulmonary alveolar proteinosis; sPAP = secondary pulmonary alveolar proteinosis.
